# Following changes in brain structure and function with multimodal MRI in a year-long prospective study on the development of Type 2 diabetes

**DOI:** 10.3389/fradi.2025.1510850

**Published:** 2025-02-13

**Authors:** Yingjie Wang, Richard Ortiz, Arnold Chang, Taufiq Nasseef, Natalia Rubalcaba, Chandler Munson, Ashley Ghaw, Shreyas Balaji, Yeani Kwon, Deepti Athreya, Shruti Kedharnath, Praveen P. Kulkarni, Craig F. Ferris

**Affiliations:** ^1^ Center for Translational NeuroImaging, Northeastern University, Boston, MA, United States; ^2^ Department of Chemistry and Biochemistry, New Mexico State University, Las Cruces, NM, United States; ^3^ Department of Mathematics, College of Science and Humanity Studies, Prince Sattam Bin Abdulaziz University, Riyadh, Saudi; ^4^ Department of Psychology and Pharmaceutical Sciences, Northeastern University, Boston, MA, United States

**Keywords:** diffusion weighted imaging, voxel based morphometry, glucose tolerance, resting state functional connectivity, cerebellum

## Abstract

**Aims:**

To follow disease progression in a rat model of Type 2 diabetes using multimodal MRI to assess changes in brain structure and function.

**Material and methods:**

Female rats (*n* = 20) were fed a high fat/high fructose diet or lab chow starting at 90 days of age. Diet fed rats were given streptozotocin to compromise pancreatic beta cells, while chow fed controls received vehicle. At intervals of 3, 6, 9, and 12 months, rats were tested for changes in behavior and sensitivity to pain. Brain structure and function were assessed using voxel based morphometry, diffusion weighted imaging and functional connectivity.

**Results:**

Diet fed rats presented with elevated plasma glucose levels as early as 3 months and a significant gain in weight by 6 months as compared to controls. There were no significant changes in cognitive or motor behavior over the yearlong study but there was a significant increase in sensitivity to peripheral pain in diet fed rats. There were region specific decreases in brain volume e.g., basal ganglia, thalamus and brainstem in diet fed rats. These same regions showed elevated measures of water diffusivity evidence of putative vasogenic edema. By 6 months, widespread hyperconnectivity was observed across multiple brain regions. By 12 months, only the cerebellum and hippocampus showed increased connectivity, while the hypothalamus showed decreased connectivity in diet fed rats.

**Conclusions:**

Noninvasive multimodal MRI identified site specific changes in brain structure and function in a yearlong longitudinal study of Type 2 diabetes in rats. The identified diabetic-induced neuropathological sites may serve as biomarkers for evaluating the efficacy of novel therapeutics.

## Introduction

Diabetes, a severe metabolic disorder, was estimated to impact around 30 million individuals in the United States in 2016, and it is anticipated that the prevalence will surpass 54.9 million Americans by 2030 (1, 2). The condition is categorized into two main types: type 1 diabetes (T1D) and type 2 diabetes (T2D). T1D is characterized by the destruction of pancreatic beta cells, leading to insulin deficiency, while T2D typically results from a combination of peripheral insulin resistance and dysfunctional insulin secretion by pancreatic beta cells (3). T2D is more prevalent in the U.S., constituting 90%–95% of all diabetes cases (1). The prevelance of T2D is enhanced by diet. For example, adopting a Western-style diet characterized by features such as a high fat/high fructose content (HF/HFr) and low vitamin levels, combined with a sedentary lifestyle, can contribute to the development of metabolic syndrome. This cluster of conditions encompasses elevated blood pressure, high blood sugar, obesity—particularly an accumulation of excess body fat around the waist—and abnormal cholesterol or triglyceride levels. The presence of metabolic syndrome heightens the risk of developing T2D and cardiovascular disease, as evidenced by numerous studies (4–7). This trend has fueled a global obesity epidemic, with 42% of Americans currently classified as overweight, according to The Center For Disease Control And Prevention in 2022. Obesity, a consequence of this dietary and lifestyle pattern, can induce a range of physiological changes, including heightened inflammation both in the peripheral tissues and the brain (8).

The systemic pathology of T2D affects various body functions, including the brain, leading to significant impacts on cognition and behavior with disease progression and aging (9). Magnetic resonance imaging (MRI) studies have reported abnormalities in cerebral macrostructure and microstructure in individuals with T2D, such as cortical atrophy, regional reductions in brain volume, structural deformities in cerebral gray matter, increased white matter lesions, and changes in blood-brain barrier permeability (10–14). Numerous studies utilizing multimodal MRI, such as voxel-based morphometry (VBM), diffusion-weighted imaging (DWI), and resting-state BOLD functional MRI (rsFC), aim to comprehend brain structure and function in T2D patients for a better understanding of disease progression and the prognosis for cognitive decline (15).

While numerous clinical MRI studies exist, there is a notable absence of research on animal models of T2D utilizing imaging techniques commonly employed in clinical settings. Only two studies have been found in this regard—one investigating ischemic vascular damage and axonal density after stroke in the high-fat diet, streptozotocin-treated Wistar rat (HFD/STZ) (16), and another in the TALLYHO/JngJ (TH) mouse, exploring the correlation between white matter connectivity using DWI and compulsive behavior. This highlights the untapped potential of non-invasive animal imaging for tracking disease progression using the same modalities as in clinical practice (17).

In a previous study (18, 19) we used multimodal MRI to characterize the neuropathology of the obese Bio-Breeding Zucker diabetic (BBZDR/Wor) rat, an established T2D model (20). We judged the model to be inappropriate for translational research as the data from MRI did not agree with that reported in the clinic. In this study we used streptozotocin, high fat, high fructose diet (HF/HFr STZ) to induced T2D in rats (21, 22). With this model, we were able to do a prospective, longitudinal study on disease progression in diabetes starting with normal healthy female rats.

## Materials and methods

### Animals

Twenty adult female Sprague Dawley rats 80–85 days of age, weighting ca 250 gm were obtained from Charles River Laboratory (Wilmington. MA). Animals were housed in Plexiglas cages in pairs and maintained in ambient temperature (22–24°C) on a 12:12 reversed light-dark cycle (lights off at 07:00 h). All experiments were conducted under dim red illumination between 10:00 h and 18:00 h to avoid the transitions between the L-D dark cycles. Using the means and SDs from previous studies from our lab (23–25), we calculated and estimated a minimum sample size of 10 subjects for each experimental group using a two-tailed test, with an alpha of 0.05, beta of 0.10, and power of 0.90. We studied females because of the dearth of literature on this sex in preclinical studies on T2D (26). All animals were cared for in accordance with the NIH Guide to the Care and Use of Laboratory Animals. Methods and procedures used in this study were pre-approved by the Northeastern University Institutional Animal Care and Use Committee, protocol # 21-1241R. The protocols used in this study followed the ARRIVE guidelines for reporting *in vivo* experiments in animal research (27). Animals were monitored daily over the duration of the study for general health, food, and water consumption. A 15% loss in body weight was set as a humane endpoint.

### Treatment

**Figure 1:**
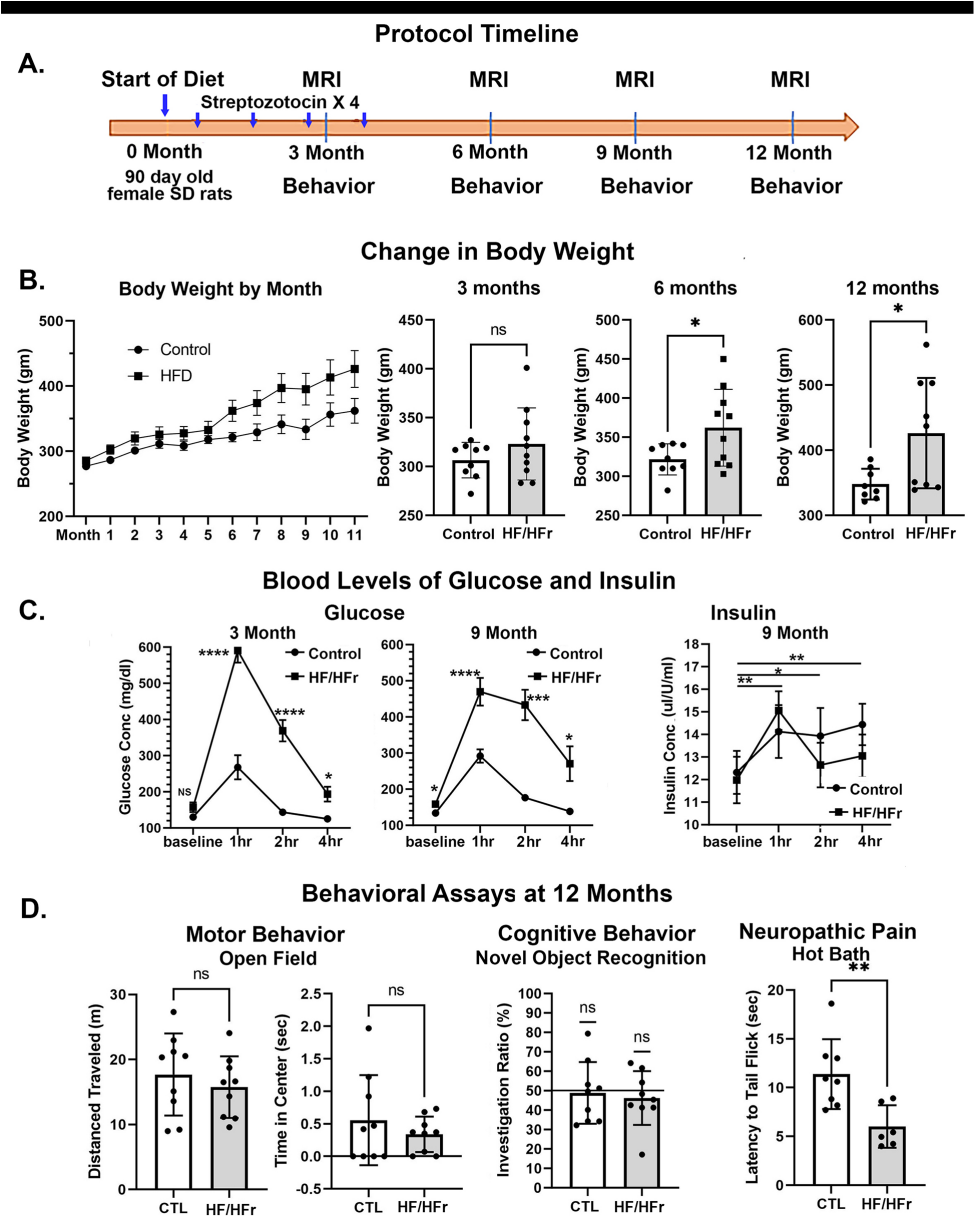
Shown in row **(A)** is the protocol timeline noting the approximate time and intervals for streptozotocin injection (blue arrows). Row **(B)** shows a time course for body weights by month and dot (subjects) plots and bar graphs with mean ± SD at 3, 6 and 12 months. Row **(C)** shows hourly time course for glucose and insulin levels collected at 3 and 9 months. Row **(D)** are dot plots and bar graphs for motor behavior in the open field, cognitive behavior in a novel object recognition assay and latency to tail flick in a hot water test of pain. *p < 0.05; **p < 0.01; ***p < 0.001; ****p < 0.0001.

The treatment protocol and timeline are shown in Figure 1A. The 20 rats were divided into two separate groups. The first group was put on a diet of standard rat chow, the second on a (HF/HFr) diet. The diet (TD.05482 High Fat Fructose Diet, casein 210 g/kg, fructose 460 g/kg, lard 255 g/kg) was supplied by Inotiv (West Lafayette, IN). Rats started on diets at 90 days of age. Food and water were provided *ad libitum.* Diabetes was induced by intraperitoneally injection of four times of STZ (25 mg/kg) given at three week intervals. STZ has a preferential toxicity towards beta cells of the pancreas.

### Body weights, blood glucose and insulin levels

The weights of all the animals were recorded weekly. Blood samples were collected at 3 and 9 months to assay glucose and insulin levels after a glucose challenge of 2 gm/kg IP after an overnight fast. Blood was drawn from the saphenous vein at time 0 (baseline) followed by 1 h, 2 h and 4 h post IP glucose injection. Blood glucose level at each time point was detected by glucometer and strips which were obtained frum Accu-chek. Insulin level was assayed using an Invitrogen- Insulin Rat Elisa Kit (Thermo Fisher Scientific).

### Behavior

#### Open field

Testing in the OF was used to assess anxiety, exploratory behaviors, and locomotor ability. The procedure has been described in previous studies (28, 29). All behavior was video-taped, and the amount of time spent in the center as well as the total distance traveled in the open field were determined using ANY-maze tracking software (Stoelting, Wood Dale, IL, USA). Each measure for the two experimental groups was compared with a *t*-test using GraphPad Prism version 9.1.2 (GraphPad Software, San Diego, California USA).

#### Novel object recognition

A novel object recognition test (NOR) was used to assess episodic learning and memory related to stimulus recognition as previously described (30, 31). The rats were video recorded and analyzed using manual methods by experimenters that were blind to treatment condition and verified with automated scoring using ANY-maze software. Investigation ratios (IR = time spent investigating the novel object/time spent investigating both objects) were assessed using single-sample, two-tailed *t*-tests, and performance was compared to chance (i.e., IR = 0.5). An investigation ratio significantly greater than 0.5 indicates that the rats were spending more time with the novel object. Conversely, a ratio significantly smaller than chance indicates a preference for the familiar object. Analysis was performed with GraphPad Prism.

### Imaging

#### Scanning setup

Each day of imaging had a mix of control and HF/HFr STZ rats known by all the investigators. Imaging sessions were conducted using a Bruker Biospec 7.0 T/20-cm scanner (Bruker, Billerica, MA, USA). Radio frequency signals were sent and received with a quadrature volume coil built into the animal restrainer (Ekam Imaging, Boston MA USA). The design of the restraining system included a padded head support obviating the need for ear bars, helping to reduce discomfort while minimizing motion artifact. All rats were imaged under 1%–2% isoflurane while keeping a respiratory rate of 40–50 breathes/min.

#### Voxel-Based morphometry

Imaging parameters: Rapid acquisition, relaxation enhanced (RARE) pulse sequence (RARE factor 8) with following parameters; 40 slices of 0.7 mm thickness; field of view [FOV] 30 × 30 mm; in-plane resolution 256 × 256 mm; repetition time [TR] 3,310 msec; effective echo time [TE] 36 msec; NEX 3; 6 min 14 s acquisition time. A 3D MRI Rat Brain Atlas© (2012 Ekam Solutions LLC, Boston, MA USA) was used to calculate brain volume, and registered the standard structural rat template image onto high resolution T2-weighted images for each subject using a non-linear registration method implemented by Unix based software package Deformable Registration via Attribute Matching and Mutual-Saliency Weighting (DRAMMS; https://www.cbica.upenn.edu/sbia/software/dramms/index.html). The atlas (image size 256 × 256 × 63) (H × W × D) was then warped from the standard space into the subject image space (image size 256 × 256 × 40) (H × W × D) using the deformation obtained from the above step using nearest-neighbor interpolation method. In the volumetric analysis, each brain region was therefore segmented, and the volume values were extracted for 173 ROIs, calculated by multiplying unit volume of voxel in mm (3) by the number of voxels using an in-house MATLAB script. To account for different brain sizes, all ROI volumes were normalized by dividing each subject's ROI volume by their total brain volume (31–33).

#### Diffusion weighted imaging—quantitative anisotropy

A detailed description of the methods using DWI for measures of edema and changes in gray matter microarchitecture from our laboratory are published (25, 34–36). The organization of brain areas into brain regions for the final analysis of apparent diffusion coefficient (ADC) and fractional anisotropy (FA) values are provided in Supplementary Data Excel Table 1.

#### Resting state functional connectivity

Scans were collected using a spin-echo triple-shot EPI sequence (imaging parameters: matrix size = 96 × 96 × 20 (H × W × D), TR/TE = 1,000/15 msec, voxel size = 0.312 × 0.312 × 1.2 mm, slice thickness = 1.2 mm, with 200 repetitions, time of acquisition 15 min. For preprocessing, we utilized a combination of various software tools, including Analysis of Functional NeuroImages (AFNI_17.1.12), the FMRIB Software Library (FSL, v5.0.9), Deformable Registration via Attribute Matching and Mutual-Saliency Weighting (DRAMMS 1.4.1), and MATLAB. Brain tissue masks for resting-state functional images were manually delineated using 3DSlicer and applied for skull-stripping. We identified motion outliers which are data segments affected by substantial motion and recorded the corresponding time points for later regression. Large motion spikes were also detected and removed from the time-course signals. Following this step, slice timing correction was applied to account for interleaved slice acquisition order. We performed head motion correction using the six motion parameters, with the first volume serving as the reference image. Normalization involved registering functional data to the 3D MRI Rat Brain Atlas © using affine registration through DRAMMS. This atlas contains 173 annotated brain regions and was used for segmentation. After quality control, a band-pass filter (0.01 Hz to 0.1 Hz) was applied to reduce low-frequency drift effects and high-frequency physiological noise for each subject. The resulting images underwent detrending and spatial smoothing, with a full width at half maximum of 0.8 mm. Additionally, regressors, including motion outliers, the six motion parameters, the mean white matter, and cerebrospinal fluid time series, were incorporated into general linear models for nuisance regression to eliminate unwanted effects.

The region-to-region functional connectivity analysis was conducted to measure the correlations in spontaneous BOLD fluctuations. In this analysis, a network consists of nodes (brain regions of interest or ROIs) and edges (connections between regions). We averaged the voxel time series data within each node based on the residual images obtained through the nuisance regression procedure. Pearson's correlation coefficients were computed across all pairs of nodes (14,535 pairs) for each subject within both groups to assess interregional temporal correlations. The resulting *r*-values, ranging from −1 to 1, were z-transformed using Fisher's Z transform to improve their normality. We constructed 173 × 173 symmetric connectivity matrices, with each entry representing the strength of an edge. Group-level analysis was then conducted to examine functional connectivity in the experimental groups. The Z-score matrices obtained from one-group *t*-tests were clustered using the K-nearest neighbors clustering method to identify how nodes cluster together and form resting-state networks. A Z-score threshold of |Z| = 2.3 was applied to eliminate spurious or weak node connections for visualization purposes.

### Functional connectivity analysis

#### Degree centrality

We conducted all network analysis using Gephi, which is an open-source software for network analysis and visualization (37). We imported the absolute values of the symmetric connectivity matrices for both HF/HFr STZ rats and controls, treating the edges as undirected networks. Degree centrality analysis measures the number of connections that a particular node has within the entire network. Degree centrality is defined as:

$${\text{C}}_{\text{D}}(\text{j})=\overset{\text{n}}{\underset{\text{j}=1}{\sum }}\;{\text{A}}_{\text{i}j}$$

Here, “*n*” represents the total number of rows in the adjacency matrix denoted as “*A*,” and the individual elements of the matrix are indicated as “*A*_*ij*_,” which signifies the count of edges connecting nodes *i* and *j*.

#### Statistics

We conducted all statistical analysis for the graph theory assessment using GraphPad Prism. To decide whether parametric or non-parametric assumptions were appropriate for different group subregions, we performed normality tests. We used Shapiro–Wilk's tests to assess the normality assumption. Subregion degree centrality *p*-values exceeding 0.05 were considered to exhibit a normal distribution. Once the normality assumptions were confirmed, we employed paired *t*-tests to compare the degree centrality between the experimental groups in various subregions. In cases where there was evidence against the normality assumption, we conducted a non-parametric Wilcoxon signed-rank (WSR) test.

## Results

### Body weight

Shown in Figure 1B are changes in body weight over time. A two-way repeated ANOVA showed there was a significant interaction between weight × time [*F*_(8,99)_ = 31.25 *p* < 0.0001]. Rats maintained on the HF/HFr STZ protocol were significantly heavier than untreated controls feed on normal rat chow at six and nine months (*t*-test, *p* < 0.05).

### Glucose challenge: blood glucose and insulin

Shown in Figure 1C are measures of blood glucose (mean ± SE) at various time points followed a glucose challenge at 3 and 9 months post diet. At 3 and 9 month there was a significant time by treatment effect *F*_(3,9)_ = 25.30, *p* < 0.0001 and *F*(3,48) = 11.43 *p* < 0.0001, respectively. Tukey's multiple comparison *post hoc* test showed HF/HFr STZ rats had higher glucose levels than controls at all time points. At 9 months, baseline levels of glucose were significantly higher (*p* < 0.05) in HF/HFr STZ rats than controls. Figure 1C shows the elevation in blood insulin levels following a glucose challenge at 9 month post diet. Both control and HF/HFr STZ rats showed a significant effect for time [*F*_(2.028,31.09)_ = 8.941, *p* < 0.001]. *post hoc* tests showed levels of insulin were significantly elevated at each time point.

### Behavioral assays

Shown in Figure 1C are scatter plots with bar graphs (mean ± SD) for motor behavior in the open field, cognitive behavior using novel object recognition and a test for pain sensitivity using the hot bath tail flick assay at 12 months of diet. There were no significant differences in motor or cognitive behaviors at 3, 6, 9, and 12 months. Peripheral neuropathy was only tested at 12 months. There were no significant differences in distance traveled or time in the center of the open field between control and HF/HFr STZ rats. Neither was there any significant differences between experimental groups for novel object preference. However, it should be noted that neither group was better than chance (50% line) suggesting that both groups had impaired spatial memory. There was a significant decrease in tail flick latency for HF/HFr STZ rats as compared to controls (*t*-test *p* < 0.01).

### Voxel based morphometry

**Figure 2:**
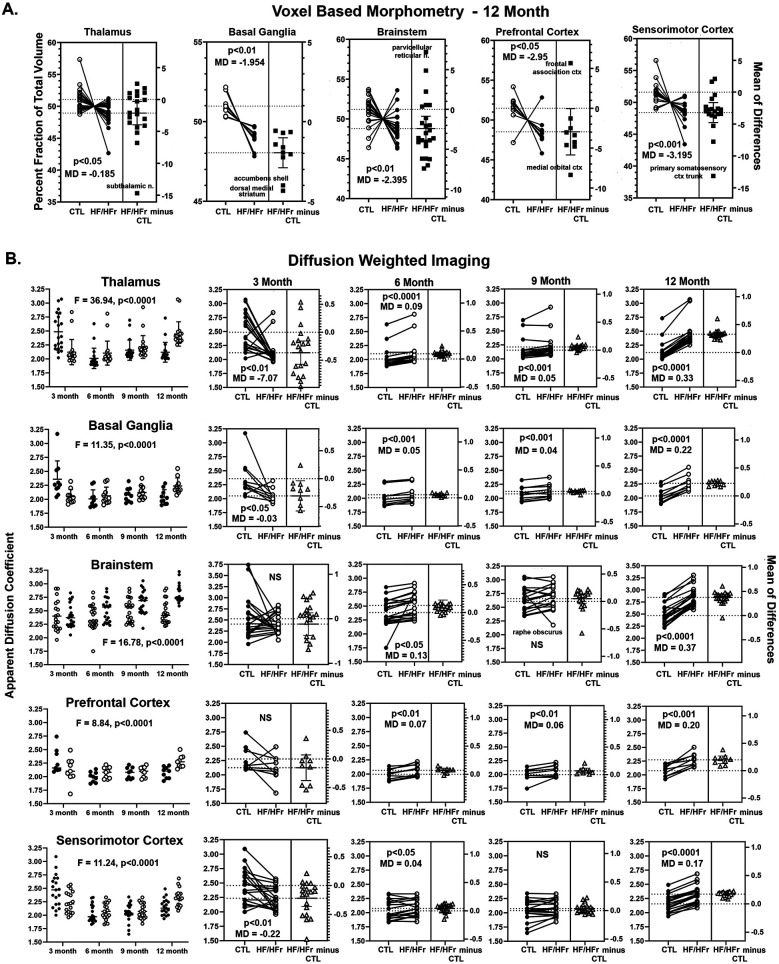
Shown in **(A)** are changes in brain volumes for different brain regions presented as a percentage fraction of the total volume. The open circle are controls (CTL) and closed circles HF/HFr STZ rats. Each circle is a specific brain area in that brain region. In all cases there was a significant decrease in brain volume with a negative median difference (MD) score included in each figure along with a *p* value. Shown in **(B)** are values for apparent diffusion coefficient from diffusion weighted imaging. The same brain regions represented in **(A)** are included in **(B)** the first column is a time course plot of controls (solid circles) and HF/HFr STZ rats (open circles) for each brain region for months 3, 6, 9, and 12 including a F value and *p* value for significance using a repeated measures two-way ANOVA. The individual results for each month shown in the row to the right together with *p*-value and MD score.

Shown in Figure 2A are the brain regions from HF/HFr STZ rats that present with significantly lower brain volumes after 12 months as compared to controls. There were no significant differences between experimental conditions for any brain regions in months 3, 6, and 9. The data are presented as the difference in the percent fraction of the total volume with open (controls) and closed (HF/HFr STZ) circles representing each brain area in that region (see Supplementary Excel File Table 1 for organization of brain regions). For example, the thalamus is comprised of 20 brain areas all of which show a decline in brain volume in HF/HFr STZ rats as compared to controls. The difference between groups is significant (two-tailed, paired *t*-test, *p* = 0.017) with a mean difference (MD) of −0.185. A scatter plot (solid squares) of the mean of differences (HF/HFr STZ minus CTL) is shown to the right for all brain areas. The brain area with the highest negative difference is the subthalamic n. The basal ganglia comprised of 10 brain areas also showed a decline in volume for each with a group significant difference (*p* = 0.001) and a mean difference of −1.954. The brain areas with the highest decrease in brain volume as shown in the scatter plot were the accumbens shell and he dorsal medial striatum. The brainstem comprised of 24 brain areas had a majority (20/24) show a decline in brain volume. The group was significantly different (*p* = 0.003) with a median differences of −2.395. The brain area with the highest increase in brain volume in the HF/HFr STZ group was the parvicellular reticular n. The prefrontal cortex comprised of eight brain areas showed a decrease in volume with the exception of the frontal association cortex. As a group, there was a significant decrease in volume (*p* = 0.047) with a median difference of −2.95. The cortex comprised of 19 brain areas all showed a decrease in brain volume that was highly significant (*p* = 0.0005) with a mean difference of −3.195. The area with the greatest mean difference was the primary somatosensory cortex representing the trunk. It should be noted that the brain regions representing the olfactory system, amygdala, hypothalamus, pons, midbrain, and cerebellum did not show a meaningful change in brain volume between controls and HF/HFr STZ rats.

### Diffusion weighted imaging

Shown in Figure 2B are time series for changes in ADC values for the same brain regions that showed significant changes in brain volume at 12 months (Figure 2A). The column on the left are dot plots (mea*n* ± SD) for each of the four time periods comparing controls and HF/HFr STZ rats for each brain areas. A two-way ANOVA showed a significant interaction between time and diet for each brain areas: Thalamus *F*_(3,114)_ = 36.94, *p* < 0.0001; Basal Ganglia *F*_(3,54)_ = 11.35, p,0.0001; Brainstem *F*_(3,119)_ = 16.78, *p* < 0.0001; Prefrontal Cortex *F*_(3,48)_ = 8.84, *p* < 0.0001 and Sensorimotor Cortex *F*_(3,108)_ = 11.24, *p* < 0.0001. Each of the individual ADC values measured over the year-long study for controls and HF/HFr STZ rats for each brain area for each brain regions are shown in the columns to the right. Shown in each is the significant group difference using a two-tailed, paired *t*-test with a mean difference (MD). A scatter plot (solid squares) of the mean of differences (HF/HFr STZ minus CTL) is shown to the right for all brain areas. At 3 months the thalamus, basal ganglia and sensorimotor cortex showed significantly lower measures of ADC in HF/HFr STZ rats on the diet. ADC values were non-significant for brainstem and prefrontal cortex. At 6 months all brain regions showed significantly greater ADC values for rats on the HF/HFr diet. At 9 months this was also true with the exception of the brainstem. At 12 months all brain regions showed significantly greater ADC values in the HF/HFr rats (*p* < 0.0001 for all brain regions but prefrontal cortex with *p* < 0.001). The mean differences for ADC values between control and diet rats were much greater at 12 months than at any other time.

Table 1 is a list of 86/173 brain areas that present with a significant difference in ADC values between control and HF/HFr STZ at 12 months of diet. Brain areas are ranked in order of significant (Ave ± SD) together with their *p*-value and omega square (Ω Sq) for effect size. In all case the ADC values are greater in HF/HFr STZ rats than controls as would be expected from Figure 2.

**Table 1:** Apparent diffusion coefficient with 12 month diabetes.

Brain area	Control		HFD		*P* val	Ω SQ
	Ave	SD	Ave	SD		
Root of trigeminal nerve	2.6	0.19	<2.9	0.11	0.001	0.698
Sub coeruleus n.	2.2	0.22	<2.7	0.21	0.002	0.577
Pontine reticular n. caudal	2.3	0.22	<2.8	0.20	0.002	0.576
Raphe magnus	2.4	0.21	<3.0	0.32	0.002	0.575
Pontine reticular n. oral	2.2	0.19	<2.6	0.20	0.003	0.554
Median raphe n.	2.3	0.20	<2.7	0.17	0.003	0.531
Prerubral field	2.1	0.20	<2.5	0.23	0.003	0.531
Parabrachial n.	2.3	0.21	<2.7	0.28	0.003	0.530
Pedunculopontine tegmentum	2.1	0.20	<2.6	0.21	0.003	0.530
Reticulotegmental n.	2.3	0.21	<2.7	0.19	0.003	0.530
Bed n. stria terminalis	2.0	0.16	<2.3	0.16	0.003	0.530
Trapezoid body	2.4	0.18	<2.9	0.27	0.003	0.530
Medial orbital ctx	2.1	0.17	<2.5	0.22	0.004	0.514
Central medial thalamic n.	2.0	0.20	<2.4	0.26	0.005	0.488
Ventromedial thalamic n.	2.0	0.18	<2.4	0.19	0.005	0.488
Motor trigeminal n.	2.2	0.19	<2.7	0.28	0.005	0.487
Intercalated amygdaloid n.	2.2	0.19	<2.8	0.37	0.005	0.487
9th cerebellar lobule	2.1	0.28	<2.6	0.28	0.005	0.467
Parvicellular reticular n.	2.4	0.17	<2.8	0.20	0.005	0.467
Ventrolateral thalamic n.	2.0	0.20	<2.3	0.24	0.006	0.447
2nd cerebellar lobule	2.3	0.27	<2.7	0.26	0.006	0.446
Precuniform n.	2.1	0.21	<2.5	0.24	0.006	0.446
Anterior thalamic nuclei	2.0	0.19	<2.4	0.24	0.006	0.445
Red n.	2.2	0.22	<2.6	0.21	0.006	0.445
Parafascicular thalamic n.	2.0	0.23	<2.4	0.27	0.007	0.427
Medial dorsal thalamic n.	2.1	0.24	<2.4	0.26	0.007	0.426
Substantia nigra compacta	2.2	0.25	<2.6	0.25	0.007	0.426
Reticular n. midbrain	2.1	0.22	<2.5	0.21	0.009	0.407
Ventral pallidum	2.0	0.14	<2.3	0.21	0.009	0.407
Periolivary n.	2.7	0.25	<3.1	0.19	0.009	0.406
Central gray	2.6	0.23	<3.0	0.28	0.009	0.405
Dentate n. cerebellum	2.5	0.23	<3.0	0.39	0.009	0.405
Ventral anterior thalamic n.	2.0	0.20	<2.3	0.24	0.009	0.405
Pontine nuclei	2.4	0.27	<2.8	0.22	0.010	0.387
Posterior thalamic n.	2.0	0.24	<2.3	0.23	0.010	0.387
Basal amygdaloid n.	2.2	0.19	<2.5	0.21	0.010	0.387
Gigantocellular reticular n.	2.4	0.21	<2.9	0.28	0.010	0.387
Zona incerta	2.1	0.20	<2.4	0.15	0.012	0.368
Anterior pretectal n.	2.1	0.31	<2.5	0.26	0.012	0.367
EXTENDED amygdala	2.0	0.19	<2.3	0.17	0.012	0.367
lateral preoptic area	2.1	0.17	<2.4	0.18	0.012	0.367
Endopiriform n.	2.0	0.11	<2.3	0.17	0.013	0.355
Principal sensory n. trigeminal	2.3	0.19	<2.7	0.27	0.014	0.349
Raphe obscurus n.	2.5	0.25	<2.9	0.29	0.015	0.334
Accumbens shell	2.0	0.16	<2.2	0.21	0.015	0.333
Anterior hypothalamic area	2.3	0.22	<2.6	0.17	0.016	0.332
Medial preoptic area	2.3	0.18	<2.6	0.17	0.016	0.332
Subthalamic n.	2.2	0.25	<2.5	0.21	0.016	0.332
Triangular septal n.	2.5	0.33	<2.8	0.19	0.016	0.332
CA3 hippocampus ventral	2.2	0.24	<2.6	0.23	0.016	0.331
Reuniens n.	2.1	0.19	<2.4	0.25	0.016	0.331
Accumbens core	1.9	0.15	<2.2	0.18	0.018	0.315
Ventral medial striatum	1.9	0.16	<2.2	0.21	0.018	0.315
Reticular n.	2.1	0.19	<2.3	0.20	0.018	0.314
4th cerebellar lobule	2.2	0.31	<2.6	0.30	0.020	0.298
Superior colliculus	2.3	0.31	<2.7	0.29	0.021	0.297
Central amygdaloid n.	2.4	0.19	<2.6	0.12	0.021	0.296
Medial pretectal area	2.4	0.58	<3.0	0.32	0.021	0.296
Ventral posterior medial thalamus	2.0	0.22	<2.3	0.21	0.021	0.296
Secondary somatosensory ctx	2.0	0.11	<2.2	0.16	0.023	0.282
Periaqueductal gray thalamus	2.5	0.21	<2.9	0.28	0.024	0.281
CA3 dorsal	2.2	0.25	<2.4	0.25	0.024	0.281
Lemniscal n.	2.3	0.25	<2.7	0.21	0.024	0.280
Lateral orbital ctx	2.0	0.15	<2.2	0.17	0.024	0.280
CA1 hippocampus ventral	2.1	0.23	<2.4	0.20	0.027	0.264
Supraoptic n.	2.4	0.28	<2.7	0.24	0.027	0.264
3rd cerebellar lobule	2.3	0.28	<2.7	0.31	0.027	0.263
Dorsomedial tegmental area	2.3	0.31	<2.6	0.26	0.027	0.263
Inferior colliculus	2.4	0.26	<2.8	0.31	0.027	0.263
Medial geniculate	2.1	0.24	<2.4	0.20	0.027	0.263
Paraflocculus cerebellum	2.2	0.18	<2.5	0.20	0.027	0.263
Posterior hypothalamic area	2.3	0.23	<2.7	0.20	0.027	0.263
Insular ctx	2.0	0.08	<2.2	0.13	0.031	0.250
Lateral dorsal thalamic n.	2.1	0.24	<2.3	0.26	0.031	0.248
Rostral piriform ctx	2.1	0.10	<2.3	0.20	0.031	0.248
Raphe linear	2.4	0.25	<2.7	0.23	0.031	0.248
Solitary tract n.	2.3	0.23	<2.7	0.34	0.034	0.237
Lateral septal n.	2.3	0.20	<2.6	0.21	0.035	0.233
8th cerebellar lobule	2.1	0.30	<2.5	0.28	0.035	0.233
Infralimbic ctx	2.0	0.19	<2.3	0.19	0.036	0.232
Ventral posteriolateral thalamus	2.0	0.20	<2.3	0.21	0.036	0.232
Primary somatosensory ctx jaw	1.9	0.09	<2.1	0.23	0.040	0.220
Cochlear n.	2.4	0.21	<2.7	0.32	0.040	0.218
Auditory ctx	2.1	0.18	<2.3	0.20	0.040	0.217
Dorsal raphe	2.8	0.53	<3.2	0.41	0.046	0.202
Paraventricular n.	2.5	0.32	<2.9	0.34	0.046	0.202

### Resting state functional connectivity

**Figure 3:**
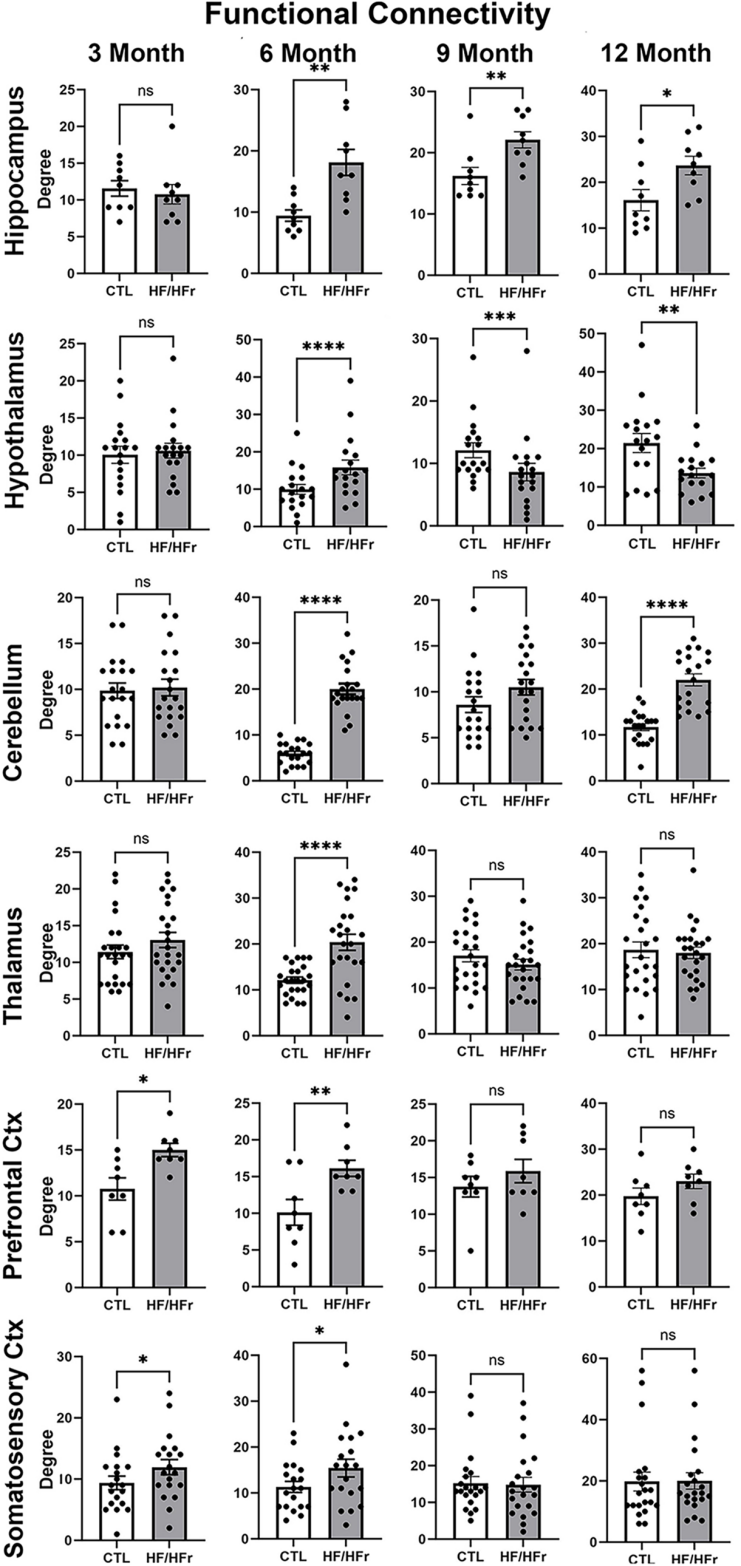
Shown are dot plots and bar graphs (mean ± SE) for the number of degrees or connections to other brain areas for control and HF/HFr STZ rats. The data are presented for six different brain regions for months 3, 6, 9, and 12. *p < 0.05; **p < 0.01; ***p < 0.001; ****p < 0.0001.

Shown in Figure 3 are dot plots and bar graphs (mean ± SEM) for changes in functional connectivity for different brain regions at 3, 6, 9, and 12 months. At 3 months from start of the HF/HFr diet there were no significant differences between control and experimental rats in degrees i.e., connections to different brain areas, in the hippocampus, hypothalamus, cerebellum, or thalamus. However, the prefrontal ctx (*p* < 0.05) and somatosensory ctx (*p* < 0.05) both presented with greater connectivity in the HF/HFr STZ group. Interestingly, by 6 months all major brain regions showed hyperconnectivity in the HF/HFr STZ rats as compared to controls. By 9 months this enhanced connectivity was lost in the cerebellum, thalamus, prefrontal ctx and somatosensory ctx. The hippocampus continued to show hyperconnectivity (*p* < 0.01) while the hypothalamus now reverted to a hypoconnected condition (*p* < 0.001). By the 12 month the connectivity for thalamus, prefrontal ctx and somatosensory ctx were unchanged with no differences between control and HF/HFr STZ groups, while the hippocampus and hypothalamus continued to show hyperconnectivity and hypoconnectivity, respectively. The cerebellum showed an interesting biphasic pattern of connectivity during the development of T2D. At 6 months it was hyperconnected, a condition that was lost by 9 months but then again hyperconnected at 12 months (*p* < 0.0001).

## Discussion

There is a paucity of MRI studies in animal models of T2D using imaging modalities commonly performed in the clinic. We know of only three such studies, one looking at ischemic vascular damage and axonal density following stroke in the high-fat diet, streptozotocin treated Wistar rat (HFD/STZ) (16), and a second in the TALLYHO/JngJ (TH) mouse correlating white matter connectivity using DWI with compulsive behavior (17). The third study was our own characterizing the obese Bio-Breeding Zucker diabetic (BBZDR/Wor) (18, 19). Hence, the major advantage of non-invasive MRI in these models—to follow the onset and progressive neuropathology associated with diabetes in the same animal with the same imaging modalities used in the clinic—has not been fully exploited. Our findings are discussed in the context of their clinical relevance and whether any of the imaging could be used as biomarkers to evaluate new therapeutics that could be tested first in animals and later in humans.

### Behavior

Dysfunctions in motor, cognitive, and emotional behavior are common in T2D. In the open field test there were no differences in the distance traveled or the time spent in the center when comparing HF/HFr STZ female rats with controls. This simple assay would suggest female rats in this model of T2D have normal motor and affective behavior. This would be in contrast to other studies that showed deficits in motor behavior in HF STZ (38) an HF/HFr STZ (21) rats. There was no difference in cognitive performance in the novel object recognition test for spatial memory between HF/HFr STZ female rats with controls in our study. It should be noted that neither group performed better than by chance. Our results are in contrast to several recent studies on HF STZ, SD males showing deficit in cognitive performance vs. controls (39–41). Pronounced deficits in learning and memory are also true in male Wistar rats on a HF/HFr STZ protocol (42).

Diabetic peripheral neuropathy stands out as the most common complication in diabetes, impacting a majority of individuals with long-term T2D (43). Most of the preclinical behavioral studies have focused on neuropathic pain and then primarily in male rats (26). In one of the few female studies, Coppey et al., exposed adult female SD rats to a HF STZ protocol for 12 wks inducing obesity and diabetes together with thermal hyperalgesia and mechanical allodynia (44). Dawane et al. reported similar results in female and male Wistar rats on the HF/HFr STZ protocol (21). Barriere and coworkers used HF/HFr male Wistar rats treated four times over 12 weeks with small doses of STZ (25 mg/kg) (22). They followed the development of T2D over 56 weeks with measures of blood chemistry, (18F)-FDG PET imaging, neuropathy, and histochemistry of various organs. Our protocol was modeled after this study but using female SD rats instead of male Wistar. Barriere et al. reported a profile of phasic metabolic changes with an initial increase in insulinemia followed by hyperglycemia and normal insulinemia at 18–42 wks with glucose challenge. The pronounced hyperglycemia and hyperinsulinemia we see at 9 months in female HF/HFr STZ, SD rats would suggest signs of early T2D. Barriere et al., reported peripheral neuropathy characterized by mechanical allodynia, but not thermal hyperalgesia as noted in our study. These differences may be due to the sex and strain of rat used in each study.

### Voxel based morphometry

A consistent observation in various imaging studies within the context of T2D is a decrease in brain volume (15). Gray matter volumes in the cortex are reduced, a finding reported in numerous studies (11, 45–50). This is also true of cerebral white matter (11, 48). The root cause of this global reduction in brain volumes remains unknown, but it is speculated to be linked to small vessel disease (51, 52). In individuals with T2D, hippocampal volumes are found to be smaller compared to age-matched controls (53). Indeed, the overall brain atrophy and decrease in gray and white matter are associated with a decline in cognitive function (45, 48, 53–55). In our study we did not find a significant change in hippocampal volume at 12 months. This may reflect the absence of any cognitive deficits. Instead, we saw a decrease in brain volumes in somatosensory/prefrontal cortices, basal ganglia and thalamus. The cortico-basal ganglia-thalamo-cortical circuit organizes and regulates information processing related to sensory and motor stimuli (56). This circuitry is also cited for its involvement in altered perception associated with schizophrenia (57). Interestingly, people diagnosed with schizophrenia have a higher prevalence of T2D as compared to the general population (58). An interesting study by Yang et al. used magnetization transfer imaging (MTI) to identify macromolecular pools of proteins in T2D patients (59). There was a decrease in MRT signal in the cortico-basal ganglia-thalamo-cortical circuit. A decrease in MRT signal is associated with loss of gray matter microarchitecture due to neuronal loss, diminished neuronal processes and altered phospholipids and membrane proteins (60).

### Diffusion weighted imaging

Diffusion-weighted imaging (DWI) serves as an indirect method for evaluating the microarchitecture of both white and gray matter. The collective findings from multiple studies consistently show alterations in microarchitecture within the frontal-temporal cortex, hippocampus, cerebellum, thalamus, and major white matter tracts (61). The cognitive decline observed in T2DM is closely linked to changes in DWI within white matter tracts, as demonstrated in numerous studies (54, 62, 63). In most cases, the typical DWI profile in individuals with T2D shows a decrease in FA and an increase in ADC. This reciprocal relationship suggests a loss of microstructural integrity, disruption in network organization and increased extracellular fluid volume possibly due to breakdown of the BBB leading to vasogenic edema. Our global voxel based analysis showed a progressive increase in ADC values with disease progression culminating in all brain regions having elevated ADC values by 12 months. The increase in ADC is consistent with the human literature across all ages and sex (61). However, in many brain regions there was also a concomitant increase in FA values which is contrary to clinical findings.

### Functional connectivity

Macpherson and colleagues conducted a comprehensive review on resting-state functional connectivity (rsFC) in T2D (64). Multiple studies consistently report a decrease in connectivity within the default mode network, specifically involving interconnections among the prefrontal cortex, parietal cortex, and hippocampus in individuals with T2D. Additionally, there is a reduction in thalamic coupling to cortical and cerebellar regions as noted by Chen et al. (65). Global organization of white matter connectivity is significantly impaired in T2D patients with cognitive dysfunction (66). There is reduced global efficiency and local nodal efficiency with fewer connections in the limbic system and basal ganglia (67). However, Zhou et al., reported an increase in nodal degree and nodal efficiency in T2D patients with mild cognitive impairment, a condition they suggest represents compensation for deficits in learning and memory (68). Contrary to much of the clinical literature, the HF/HFr STZ diabetes model used here demonstrated an unexpected impact on global connectivity. At 3 months only the prefrontal ctx and the many region-specific parts of the somatosensory ctx showed enhanced connectivity as compared to controls. By 6 months all brain regions from HF/HFr rats showed hyperconnectivity as compared to controls. This increase in connectivity may potentially be a compensatory response as noted above to underlying pathology, similar to observations in traumatic brain injury (36) or in young children with early type 1 diabetes, as reported by Saggar et al. (69). This collective increase in global voxel based connectivity may also represent an adaptive response to a metabolic disorder. By 9 months only the hippocampus retained its hyperconnectivity while the hypothalamus reversed and showed a decrease in connectivity for the HF/HFr condition. By 12 months the hippocampus and hypothalamus retained the hyper and hypo connectivity, respectively while the cerebellum became hyperconnected again as in 6 months.

### Data interpretation

In the general population, men have a slightly higher prevalence of T2D than women; although, women with T2D are at higher risk for cardiovascular complications and mortality as compared to diabetic men (70). Sex differences in T2D are understudied in animals. While men, more than women, are diagnosed in early life with T2D, women have a relatively higher risk of cardiovascular complication later in life (70). Pregnancy and gestational T2D significantly increases the risk of T2D in later life for women (71). Indeed, this was one of the few preclinical studies using an HF/HFr STZ model that focused solely on females. Moreover this is the only preclinical study that used multimodal MRI to follow the onset and development of T2D using imaging protocols commonly used in the clinic. The effort to use multimodal imaging to validate the HF/HFr STZ model of T2D as clinically relevant was partially successful. The decrease in brain volume using voxel based morphometry mirrored the human condition and disease progression. The areas that were most sensitive were associated with the cortico-basal ganglia-thalamo–cortical circuit in agreement with human image studies. The elevation in ADC values, a surrogate marker of injury to in gray matter microarchitecture complimented the VBM data and mirrored the human data but the concomitant increase in FA levels is not normally see in human T2D. The differences in global voxel based functional connectivity between controls and HF/HFr STZ rats was biphasic. During early disease progression there were no significant differences but by 6 months, HF/HFr STZ rats showed a global hyperconnectivity that reverted back to earlier levels by 9 months. The region-specific exceptions were the hippocampus which retained a level of hyperconnectivity from 6 to 12 months, the hypothalamus that went from a hyperconnected condition at 6 moths to a hypo connected state from 9 to 12 month and the cerebellum that went through two stages of hyperconnectivity at months 6 and 12. These different phases and region-specific changes in connectivity over the year-long study do not have parallels in the human literature. Much of the human literature on T2D report a decrease in functional connectivity during frank T2D with pronounced behavioral symptoms. In this study the hyperglycemia, weight gain and connectivity in HF/HFr STZ rats were significantly altered by 6 months but measures of brain atrophy with VBM and gray matter injury with DWI, the neuropathological endophenotypes of T2D, were not realized until the 12th month. There was no change in cognitive behavior but an increased sensitivity to peripheral pain. Hence some of the differences between our imaging results and those in the clinic may be due to disease severity. Another possible confound that could explain the differences in connectivity is the use of anesthesia during our data acquisition, something not found in human imaging. Structural properties of the brain measured with VBM and DWI would be less sensitive to the effects of anesthesia. The measures of brain volume and gray matter microarchitecture fit the human condition but not functional connectivity.

## Conclusion

The signs and symptom of T2D evolved over the 12 month study. Diet fed rats presented with elevated plasma glucose levels as early as 3 months and a significant gain in weight by 6 months as compared to controls. There were no significant changes in cognitive or motor behavior over the yearlong study but there was a significant increase in sensitivity to peripheral pain in diet fed rats. There were region specific decreases in brain volume e.g., basal ganglia, thalamus and brainstem in diet fed rats. These same regions showed elevated measures of water diffusivity evidence of putative vasogenic edema. By 6 months, many brain regions showed hyperconnectivity. By 12 months, only the cerebellum and hippocampus showed increased connectivity, and the hypothalamus decreased connectivity in diet fed rats. These diabetic-induced sites of putative neuropathology can be used as biomarkers to assess the efficacy of new therapeutics. Site specific increases in ADC could be used as a surrogate marker of increased blood brain barrier permeability and impending small vessel disease (34).

## Data Availability

The original contributions presented in the study are included in the article/Supplementary Material, further inquiries can be directed to the corresponding author.
